# Atypical Pharyngeal Pouch Arising Bilaterally between the Hyoid Bone and Thyroid Cartilage

**DOI:** 10.1155/2017/3515438

**Published:** 2017-04-05

**Authors:** Lawrence J. Oh, Lyndon Chan, David Veivers

**Affiliations:** ^1^Royal North Shore Hospital, St Leonards, NSW, Australia; ^2^University of Sydney, Camperdown, NSW, Australia

## Abstract

*Introduction*. Pharyngoesophageal diverticuli are a common cause of dysphagia; they are associated with various morbidities and a decreased quality of life. There are several different types of the diverticuli, and they are divided based on the anatomical location of origin relative to the cricopharyngeal muscle; these include Zenker's, Killian-Jamieson's, and Laimer's diverticula. The authors present a unique case of pharyngoesophageal diverticulum that has not been previously described.* Case Presentation*. A 65-year-old male presented with a 12-month history of dysphagia and odynophagia for solids. Barium swallow revealed bilateral moderately sized diverticuli that altered in size during the different phases of swallow. CT scan of the neck with oral contrast further identified the anatomy of the diverticuli, arising between the hyoid bone and thyroid cartilage.* Discussion*. An external transcervical approach was utilised to successfully repair the diverticuli. Subsequent cricopharyngeal spasm was treated with botulinum toxin, and the patient recovered with no ongoing symptoms. The barium swallow study is a commonly utilised initial investigation as it is easy to perform and safe and has good diagnostic value. Definitive management usually involves either endoscopic or open surgery. This case depicts a unique case of a pharyngeal diverticulum arising between the hyoid bone and thyroid cartilage.

## 1. Introduction

Pharyngoesophageal diverticulum is a disease entity that predominantly affects the elderly population and present with numerous symptoms including dysphagia, regurgitation, and obstruction, which can ultimately lead to significant morbidity. The gold standard method of initial investigation is the barium swallow [1].

The anatomical site of origin determines the type of diverticulum. By far the most common is Zenker's diverticula, characterised by a posterior outpouching originating from Killian's dehiscence of the inferior constrictor muscle, between the cricopharyngeal and thyropharyngeal muscles. The Killian-Jamieson diverticulum is much rarer and is a herniation through the anterolateral located superolateral to the longitudinal muscle of esophagus and inferior to the cricopharyngeal muscle [2, 3]. The rarest variant of pharyngeal diverticulum is often referred to as Laimer's or Laimer-Haeckerman's diverticulum. Similar to the Killian-Jamieson diverticulum, it originates between cricopharyngeus and the longitudinal muscle of the oesophagus; however, it is located posteriorly and midline, from the area termed Laimer-Haeckerman's triangle, and is covered only by the circular muscles of the oesophagus [4–6]. The various subtypes of pharyngoesophageal diverticulum are easily distinguishable with radiographic studies [2]. There have only been 3 reports of Laimer-Haeckerman's diverticuli thus far [4–6].

This case depicts a unique diverticulum that originates from between the hyoid bone and thyroid cartilage. To our knowledge, there have been no prior reports of a case matching these descriptions as it bears no relation to the cricopharyngeal muscle.

## 2. Case Presentation

**Figure 1:**
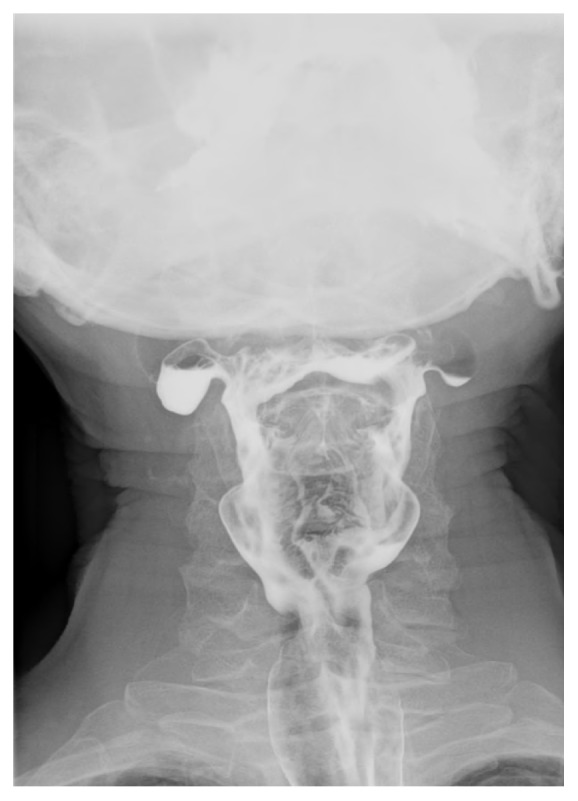
Preoperative barium swallow showing two moderate-sized diverticula.

**Figure 2:**
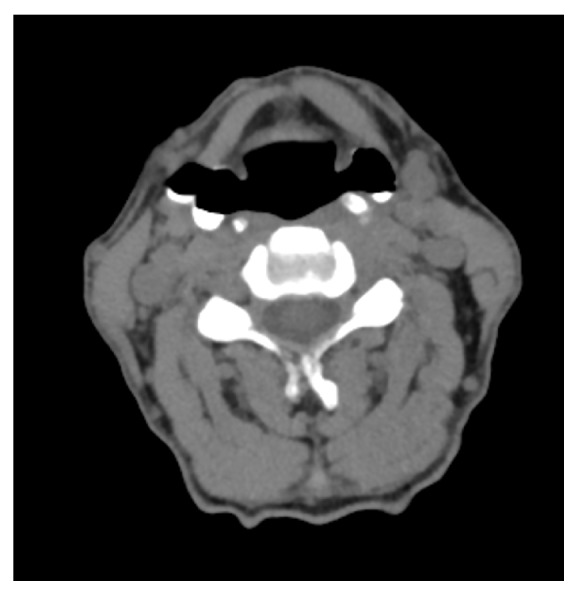
Noncontrast CT scan of the neck showing the bilateral pouches.

A 65-year-old male presented with a 12-month history of progressive dysphagia and odynophagia for solids. The patient often complained of discomfort at the level of the sternal notch when swallowing foods. Subsequently, a barium swallow was performed. Imaging revealed bilateral moderately sized diverticuli that altered in size during differing phases of swallow (Figure 1). The diverticuli were located higher and more lateral than expected for the known types of pouches, prompting a CT scan of the neck with oral contrast (Figure 2) to determine the anatomy of the diverticuli in relation to the neck structures. The CT scan portrayed the pharyngeal pouches to be 17 mm in diameter and arising between the hyoid bone and thyroid cartilage.

**Figure 3:**
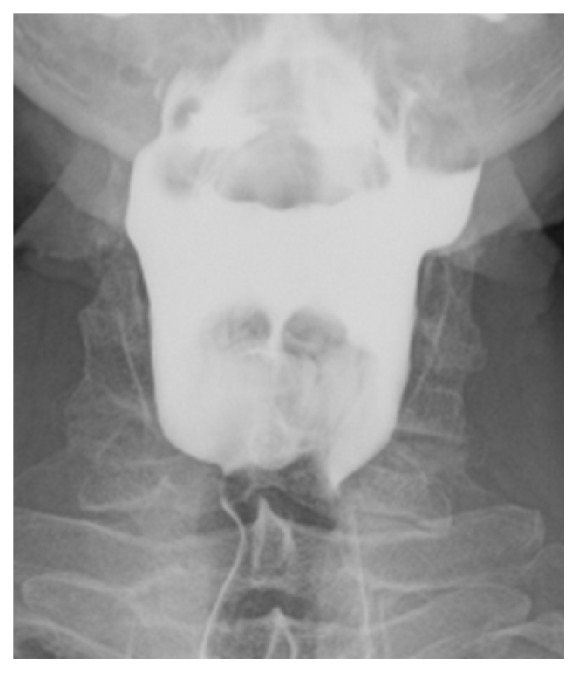
Postoperative barium swallow portraying marked improvement in the contour of the pharynx since the previous study with only mild residual ectasia of the superior lateral left wall of the pharynx.

After extensive discussion with the patient, definitive surgery was planned. Before the external transcervical approach was performed, initial pharyngoscopy to identify the mouth of the pouches and subsequent packing with ribbon gauze was performed. The mouth of the diverticulum was located on the right side, and it was packed from the pharyngeal side, just above the level of the superior border of the thyroid ala. The pouch was subsequently found to be herniating through inferior constrictor muscle and intimately related to the superior laryngeal neurovascular bundle. The right-sided diverticulum had a narrow base and was excised and closed primarily with 4-0 vicryl sutures. The left-sided lesion was broader-based and was inverted and oversewn. A nasogastric tube was placed and the patient remained in hospital postoperatively for five days until oral intake was resumed. Review at 2 months revealed significant improvement of symptoms. Unfortunately, swallowing deteriorated again at 3 months, and a repeat barium swallow was performed (Figure 3), showing that the diverticuli were well-treated but suggesting that cricopharyngeal spasm may be causing ongoing dysphagia. The patient underwent injection of botulinum toxin into the cricopharyngeus and subsequently had improvement in their symptoms.

## 3. Discussion

Pharyngoesophageal diverticuli are not true diverticuli. Their pathogenesis is explained by pulsion forces against an obstruction, usually a dysfunctional cricopharyngeus, resulting in a raised intraluminal pressure and herniation of the pharyngeal mucosa through weakened areas of the pharyngoesophagus.

This case involves bilateral diverticuli located between the hyoid bone and thyroid cartilage with no relation to the cricopharyngeal muscle. Although such a case has not been previously reported, the patient presented with the typical symptoms of dysphagia and substernal pain similar to that seen in the usual types of diverticuli [7]. A possible explanation for this pouch may lie in embryology. The pouch may have occurred in a weakness, through the thyrohyoid membrane caused by the entry of the superior neurovascular bundles and may represent a type of third branchial cleft anomaly. The fact that the symptoms only became fully controlled with subsequent treatment of the cricopharyngeus muscle probably indicates that the diverticuli were only a manifestation of the swallowing problem and not the basic cause, which is a situation that is analogous to that in Zenker's diverticulum [3].

Normally, a barium swallow is unable to distinguish between the various diverticuli [8]. Suspicion for an abnormal pouch leads to a CT scan of the neck and this subsequently aided surgical planning. Endoscopic diverticulotomy has been a well-accepted approach to surgical management of Zenker's diverticuli for the past two decades; however, Killian-Jamieson and Laimer's diverticula have usually been treated via an external approach [9, 10]. An external approach allowed for careful and accurate identification and dissection of the pouches, with preservation of the neurovascular bundle. Undavia et al. described an open transcervical approach in the management of patients with a Killian-Jamieson diverticulum, in order to preserve the recurrent laryngeal nerve and other structures and to ultimately decrease morbidity and mortality rates [2]. Kobayashi et al. describe an open excision approach along the anterior border of the sternocleidomastoid muscle with subsequent lateral retraction of the carotid sheath to visualise the diverticulum located in Laimer-Haeckerman's triangle below the cricopharyngeus muscle [5]. This external approach is reinforced by Rubesin and Levine who successfully operated on Laimer's diverticulum with Zenker's diverticulectomy without myotomy [7].

In terms of pathophysiology, the fibroadipose replacement of the cricopharyngeus muscle results in a diminished upper oesophageal sphincter, thus ultimately resulting in diminished upper oesophageal sphincter compliance and spasm. This also explains the dysphagia as constrictive myopathy can cause increases in both pharyngeal and hypopharyngeal intrabolus pressures that can result in posterior herniations through the pharyngeal wall [11]. From recent studies [12], we believe that the sternal discomfort was associated with inflammation of the mediastinum, which can be associated with motility abnormalities, including diffuse spasm.

## 4. Conclusion

This case presents a unique case of pharyngoesophageal diverticulum that has not been described in previous literature. Despite its anatomical difference from variants such as the Zenker's, Killian-Jamieson's, or Laimer's diverticuli, the patient's presenting symptoms such as dysphagia and sternal discomfort were universal. Like other non-Zenker's diverticula, we recommend an external approach to these pouches as it allows for more optimal visualisation, dissection, and preservation of important structures, thus ultimately reducing morbidity and mortality rates. Of interest is the fact that the diverticuli were probably a manifestation of a broader pharyngeal swallowing disorder, the full resolution of which occurred once incoordination at the cricopharyngeal level was addressed.
